# Analysis of clinical characteristics, prognosis and influencing factors in patients with bronchiectasis-chronic obstructive pulmonary disease overlap syndrome: A prospective study for more than five years

**DOI:** 10.7189/jogh.14.04129

**Published:** 2024-06-28

**Authors:** Zhongshang Dai, Yanjun Zhong, Yanan Cui, Yiming Ma, Huihui Zeng, Yan Chen

**Affiliations:** 1 Department of Infectious Diseases, Second Xiangya Hospital, Central South University, Changsha, China; 2 Department of Respiratory and Critical Care Medicine, Second Xiangya Hospital, Central South University, Changsha, China; 3 Research Unit of Respiratory Disease, Central South University, Changsha, China; 4 Clinical Medical Research Center for Respiratory and Critical Care Medicine in Changsha China; 5 Diagnosis and Treatment Center of Respiratory Disease, Central South University, Changsha, China

## Abstract

**Background:**

Considering the large population of bronchiectasis and chronic obstructive pulmonary disease (COPD) patients in China, we aimed to conduct a thorough analysis that investigates the clinical characteristics and prognosis of bronchiectasis-COPD overlap syndrome (BCOS). Further, we aimed to explore factors associated with acute exacerbation and death in BCOS, which may be of value in its early diagnosis and intervention.

**Methods:**

We recruited inpatients with COPD from the second Xiangya Hospital of Central South University in China in August 2016, with follow-up until March 2022. Patients in the BCOS group had to meet the criteria for diagnosing bronchiectasis. We used self-completion questionnaires, clinical records, and self-reported data as primary data collection methods. We used Kaplan-Meier survival analyses and Cox proportional hazard models to assess the risk of severe acute exacerbation and death for BCOS during the follow-up period.

**Results:**

A total of 875 patients were included and followed up. Patients in the BCOS group had more females, fewer smokers, lower discharge COPD assessment test (CAT) scores, lower forced vital capacity (FVC), a higher likelihood of co-occurring active tuberculosis, higher levels of eosinophils and inflammatory markers, and a higher rate of positive sputum cultures for *Pseudomonas aeruginosa* than patients in the COPD-only group. Patients in the acute exacerbation group (AE+) were found to have lower body mass index (BMI), more frequent acute exacerbations, higher modified Medical Research Council (mMRC) dyspnoea grade on admission, higher inflammatory markers, lower FVC, higher rates of using inhaled bronchodilators, and higher rates of both positive and *Pseudomonas aeruginosa* positive sputum cultures. Patients in the ‘death’ group were older, had a lower BMI, had spent longer time in the hospital, had higher mMRC dyspnoea grade and CAT scores upon admission and discharge, had higher levels of inflammatory markers, lower rates of using inhaled bronchodilators, were more likely to have a combination of pulmonary heart disease and obsolete pulmonary tuberculosis, as well as a higher rate of fungus-positive sputum cultures. Both erythrocyte sedimentation rate at baseline and *Pseudomonas aeruginosa* culture positivity were confirmed as independent predictors of severe acute exacerbation in multivariate analysis during the years of follow-up. Fungus culture positivity baseline blood urea nitrogen, baseline lymphocyte count, comorbidities with obsolete pulmonary tuberculosis and comorbidities with pulmonary heart disease were verified as independent predictors of death in multivariate analysis during the years of follow-up. Kaplan-Meier curves under survival analysis demonstrated no statistically significant difference in mortality between the COPD and the BCOS groups at the full one, two, and three years of follow-up.

**Conclusions:**

Patients with BCOS present with reduced lung function, increased susceptibility to different complications, elevated blood eosinophils and inflammatory markers, and elevated rates of positive *Pseudomonas aeruginosa* cultures. These distinctive markers are linked to a greater risk of severe acute exacerbations and mortality.

Chronic obstructive pulmonary disease (COPD) is a global public health challenge due to its high prevalence and related mortality [1]. According to the 2017 Global Burden of Disease report, COPD is the third leading cause of death worldwide, with approximately three million deaths each year [2]. The most recent China Pulmonary Health study reported that the overall prevalence of spirometry-defined COPD was 13.7% among the general Chinese population aged ≥40 years, and the estimated total number of individuals was 99.9 million [3]. Bronchiectasis is a long-term respiratory condition characterised by persistent airway infection and recurrent exacerbations in the presence of structurally abnormal bronchi [4]. Bronchiectasis is diagnosed with airway dilatation and airway wall thickening on imaging (usually computed tomography) and is a structural diagnosis. Several epidemiological surveys from Europe have suggested that the incidence rate of bronchiectasis is increasing in recent years [4–6]. With the widespread use of high-resolution chest computed tomography in COPD assessment, many COPD patients have been found to have concurrent bronchiectasis. In summary, bronchiectasis and COPD may co-exist as an overlapping syndrome – bronchiectasis-COPD overlap syndrome (BCOS) [7]. However, few recent, comprehensive data exist on the epidemiology and associated mortality of BCOS in China.

Foreign studies have shown that the proportion of COPD patients with bronchiectasis ranges from 25.6–69.0% [8–11]. However, the overlap between COPD and bronchiectasis is a neglected research field, almost covered by few clinical practice guidelines. A meta-analysis of six clinical observational studies found that BCOS occurred more often in male patients with a longer smoking history. BCOS patients had greater daily sputum production, more frequent acute exacerbation, poorer lung function, higher levels of inflammatory biomarkers, more chronic colonisation of potentially pathogenic bacteria, and higher rates of *Pseudomonas aeruginosa* isolation [9]. However, there have been no studies on BCOS’s clinical characteristics and prognosis and the factors influencing it in patients in China. Considering the large population of COPD and bronchiectasis patients in China, we aimed to conduct a comprehensive analysis that investigates the clinical characteristics and prognosis of BCOS patients and to explore factors associated with acute exacerbation and death in BCOS, which may be of value in its early diagnosis and intervention.

## METHODS

### Ethical approval

This study was approved by the Ethics Committee of the Second Xiangya Hospital of Central South University (number ChiCTR-POC-17010431) and conducted following the Declaration of Helsinki. We obtained written informed consent from all study participants. All methods, including the diagnosis of COPD and spirometry test, were performed following COPD guidelines and regulations [12].

### Study population

This real-world, observational, and prospective study recruited inpatients with COPD from the Second Xiangya Hospital of Central South University from August 2016 to March 2022. All recruited participants had a clear diagnosis of COPD according to the Global Initiative for Chronic Obstructive Lung Disease 2019; based on the persistent airflow limitation defined as a ratio of post-bronchodilator forced expiratory volume in one second (FEV1) to forced vital capacity (FVC)<70% [12]. Patients in the BCOS group had to meet the criteria for diagnosing bronchiectasis. Making the diagnosis of bronchiectasis requires that both symptoms (cough with sputum production, dyspnoea and wheezing) and radiological features (bronchial dilatation concerning the accompanying pulmonary artery (signet ring sign), lack of tapering of bronchi, and identification of bronchi within one cm of the pleural surface) are present [13,14]. Patients were excluded if they did not have a confirmed spirometry-verified COPD, had a primary diagnosis of other respiratory diseases other than COPD, or had missing data.

### Study design

We obtained the following information at the time of the initial visit: age, gender, body mass index (BMI), smoking history, time of first diagnosis, hospitalisation days, pulmonary function, number and severity of COPD exacerbation in the previous year, COPD assessment test (CAT) score, modified Medical Research Council (mMRC) dyspnoea grade, comorbidities, current treatment regimen, blood routine, inflammatory markers, hepatic and renal function, blood gas analysis, immunoglobulin and sputum culture. The diagnostic criteria for combined hypertension were blood pressure exceeding 140/90 mm Hg thrice on a different day. The definition of combined active pulmonary tuberculosis began with a positive acid-fast bacillus smear or a positive culture for mycobacterium tuberculosis [12]. Self-completion questionnaires, clinical records, and self-reported data were primary data collection methods. All pulmonary function tests were performed by laboratory technicians at the pulmonary clinics. Trained research assistants collected the data, and the variables were checked by trained doctors [15].

Exacerbation was defined as acute worsening of respiratory symptoms that required a short-acting bronchodilator only (mild), short-acting bronchodilator plus antibiotic and/or oral corticosteroid (moderate), or hospitalisation/emergency department visit (severe) [15,16]. We followed up with all patients for >12 months. Research assistants collected data on COPD exacerbations in the first and last year of follow-up and on all-cause deaths throughout the follow-up period via a telephone consultation with patients and their relatives, interviews in outpatient clinics, or through a review of medical records provided by patients. The primary outcomes were hospitalisation for COPD exacerbation and mortality.

### Statistical analysis

We analysed data using SPSS, version 21.0 (IBM, Armonk, New York, USA) and Free Statistics, version 1.7.1 (Beijing, China). The data in this study were non-normally distributed after the normality test. Descriptive data without normal distribution were expressed as medians (the 25th and 75th percentile), and frequencies were expressed as numbers (percentages). We compared groups using the χ^2^ test or Fisher exact test for categorical variables. We compared continuous variables using a one-way analysis of variance, the Mann-Whitney U-test, or the *t* test.

We used Kaplan-Meier survival analyses and Cox proportional hazard models to assess the risk of hospitalisation for BCOS exacerbation, as well as death during the follow-up period. Further, we used multivariate Cox regression models to evaluate the risk of exacerbations during the follow-up period. We adjusted the potential confounding factors with a *P*-value of  0.10 between groups in baseline characteristic analyses in the models (age and gender were included because of their significant clinical relevance, but variables related to grouping were not considered to avoid potential collinearity). Statistical significance was set at *P* < 0.05.

## RESULTS

### Comparisons of clinical characteristics between COPD and BCOS groups

**Figure 1:**
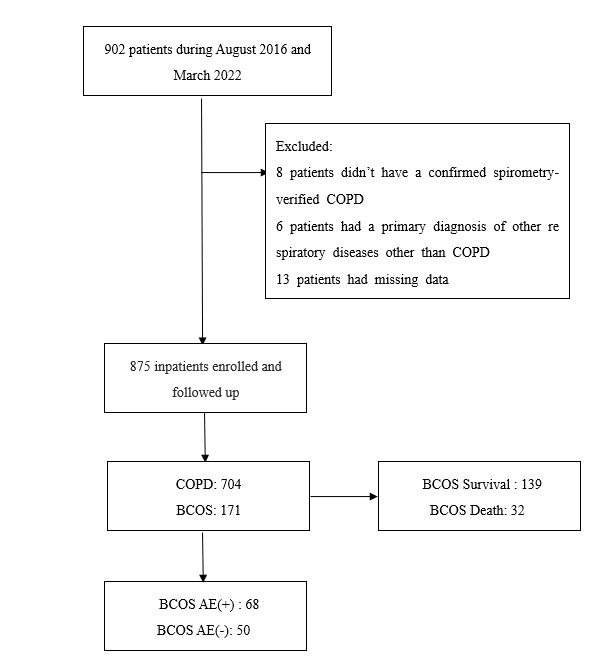
Flow diagram. COPD – chronic obstructive pulmonary disease, BCOS – bronchiectasis-chronic obstructive pulmonary disease overlap syndrome, AE – acute exacerbation.

Of the original cohort including 902 inpatients, 27 patients were excluded. Eight patients had no spirometry results, six patients had a primary diagnosis of other respiratory diseases other than COPD, and 13 patients had missing data. 875 patients meeting the study criteria were included and followed up. Initially, we divided patients into the COPD and BCOS groups for comparison of clinical characteristics. During the follow-up process, we divided patients in the BCOS group into acute exacerbation (AE+) and no acute exacerbation (AE−) groups, depending on whether severe acute exacerbation occurred. Further, we divided patients in the BCOS group into ‘survival’ and ‘death’ groups based on whether they experienced death (**Figure 1**).

Males constituted 89.1% of the respondent group, while 10.9% were females. Most participants (82.7%) were previous or current smokers. Of the included patients, 15.4% were AE+, 39.6% were AE−, and 45.0% had two or more acute exacerbations during the previous year. Nearly one-third of the participants had combined hypertension, and a vast majority (98.4%) did not have combined active pulmonary tuberculosis.

In the COPD and BCOS groups, there was no significant difference in BMI, first diagnosis before 50 years old, hospitalisation days, times of exacerbations in the past 12 months, CAT score for admission, improvements in mMRC dyspnoea grade, forced expiratory volume in one second percentage predicted (FEV1%), combined diabetes mellitus, combined obsolete pulmonary tuberculosis, combined coronary artery disease and combined pulmonary heart disease. Compared to the COPD group, patients in the BCOS group exhibited statistically significant differences (*P* < 0.05) in terms of a higher proportion of females, fewer patients with a history of smoking and a lower smoking index, lower discharge CAT scores, a more significant improvement in CAT scores, lower FVC, higher FEV1/FVC, lower proportion of combined hypertension and arrhythmia, and a higher proportion of combined active tuberculosis (**Table 1**). Upon analysing the laboratory data of patients with COPD and BCOS, we discovered that, in contrast to the COPD group, patients in the BCOS group had significantly higher levels of platelet count (PLT), eosinophil count, erythrocyte sedimentation rate (ESR), C-reactive protein (CRP), globulin (GLO), immunoglobulin A (IgA), and immunoglobulin G (IgG), and lower levels of creatinine, and blood urea nitrogen (BUN). Furthermore, the BCOS group exhibited a greater sputum culture positivity rate than the COPD group. Notably, there was a statistically significant difference (*P* < 0.001) in the positivity rate of *Pseudomonas aeruginosa* in sputum culture (19.4% and 2.4%). There was no statistically significant difference in the comparison of blood gas analysis between the two groups (**Table 2**).

**Table 1:** Comparison of baseline characteristics between COPD and BCOS group*

Characteristics	Total (n = 875)	COPD (n = 704)	BCOS (n = 171)	*P-*value
Age in years	68 (62–75)	69 (63–75)	67 (61–72)	0.012
Male, n (%)	780 (89.1)	647 (91.9)	133 (77.8)	<0.001
BMI (kg/m^2^)	21.3 (18.7–24.2)	21.4 (18.8–24.4)	20.9 (18.6–23.5)	0.186
Previous/current smokers, n (%)	724 (82.7)	614 (87.2)	110 (64.3)	<0.001
Smocking pack-years	38 (15–50)	40 (20–55)	20 (0–40)	<0.001
First diagnosis before 50 y old, n (%)	69 (7.9)	58 (8.2)	11 (6.4)	0.432
Hospitalisation days	9 (8–12)	9 (8–12)	10 (8–12)	0.348
Times of exacerbations in the past 12 mo, n (%)				2.013
*0*	125 (15.4)	104 (15.9)	21 (13.5)	
*1*	321 (39.6)	252 (38.4)	69 (44.5)	
*≥2*	365 (45.0)	300 (45.7)	65 (41.9)	
CAT score for admission	23 (18–28)	23 (18–28)	22 (17–27)	0.652
mMRC dyspnoea grade for admission	3 (2–4)	3 (2–4)	3 (2–4)	0.021
Improvements in CAT score	6 (2–10)	5 (2–9)	7 (3–12)	0.030
Improvements in mMRC dyspnoea grade	1 (0–1)	1 (0–1)	1 (0–1)	0.840
CAT score for discharge	16 (11–22)	16 (11–22)	14 (10–20)	0.032
mMRC dyspnoea grade for discharge	16 (11–22)	16 (11–22)	14 (10–20)	0.032
FEV1% predicted	34.5 (25.0–47.2)	34.9 (25.0–47.6)	33.4 (26.4–44.7)	0.406
FVC (L)	2.11 (1.67–2.59)	2.16 (1.73–2.63)	1.94 (1.43–2.38)	<0.001
FEV1/FVC	39.2 (31.1–51.0)	38.0 (30.7–48.7)	43.4 (34.2–60.0)	<0.001
Comorbidities, n (%)				
*Hypertension*	314 (35.9)	265 (37.6)	49 (28.7)	0.028
*Diabetes mellitus*	108 (12.3)	88 (12.5)	20 (11.7)	0.774
*Obsolete pulmonary tuberculosis*	261 (29.8)	209 (29.7)	52 (30.4)	0.853
*Active pulmonary tuberculosis*	14 (1.6)	8 (1.1)	6 (3.5)	0.027
*Coronary artery disease*	149 (17.0)	126 (17.9)	23 (13.5)	0.165
*Arrhythmia*	101 (11.5)	90 (12.8)	11 (6.4)	0.020
*Pulmonary heart disease*	189 (21.6)	157 (22.3)	32 (18.7)	0.306

BCOS – bronchiectasis-chronic obstructive pulmonary disease overlap syndrome, BMI – body mass index, CAT – chronic obstructive pulmonary disease assessment test, COPD – chronic obstructive pulmonary disease, FEV1 – forced expiratory volume in one second, FVC – forced vital capacity, IQR – interquartile range, MD – median, mMRC – modified Medical Research Council

*Presented as MD (IQR) unless specified otherwise.

**Table 2:** Comparison of laboratory examination between COPD and BCOS groups*

Laboratory examinations	Total (n = 875)	COPD (n = 704)	BCOS (n = 171)	*P*-value
Blood routine				
*WBC ( × 10^9^/L)*	7.07 (5.66–9.21)	7.08 (5.68–9.23)	7.04 (5.51–9.07)	0.975
*HB (g/L)*	131 (118–141)	131 (118–142)	129 (117–140)	0.303
*PLT ( × 10^9^/L)*	208 (163–267)	204 (162–260)	226 (175–289)	0.1010
*Neutrophils ( × 10^9^/L)*	5.13 (3.94–7.11)	5.13 (3.95–7.07)	5.16 (3.94–7.15)	0.891
*Lymphocytes ( × 10^9^/L)*	1.14 (0.81–1.47)	1.14 (0.80–1.49)	1.16 (0.87–1.42)	0.619
*Eosinophils ( × 10^9^/L)*	0.12 (0.05–0.22)	0.11 (0.05–0.21)	0.15 (0.06–0.25)	0.019
*Eosinophils ≥0.3 × 10^9^/L, n (%)*	142 (16.3)	105 (14.9)	37 (21.6)	0.032
*LNR*	0.22 (0.14–0.33)	0.22 (0.13–0.33)	0.23 (0.14–0.32)	0.509
Inflammatory markers				
*CRP (mg/L)*	8.65 (3.48–25.80)	8.46 (3.38–25.40)	9.17 (3.91–26.80)	0.258
*ESR (mm/h)*	18 (7–40)	17 (7–36)	25 (8–48)	0.029
*PCT (mg/L)*	0.10 (0.05–0.13)	0.10 (0.05–0.14)	0.10 (0.05–0.12)	<0.001
Hepatic and renal function				
*ALB (g/L)*	34.50 (31.90–37.60)	34.60 (32.00–37.80)	34.20 (31.30–37.20)	0.117
*GLO (g/L)*	26.30 (23.40–30.10)	26.00 (23.00–29.60)	27.60 (24.90–31.10)	<0.001
*Creatinine (μmol/L)*	68.20 (57.70–81.90)	68.70 (58.30–82.50)	65.40 (56.00–76.70)	0.028
*BUN (mmol/L)*	5.56 (4.38–7.19)	5.64 (4.48–7.24)	5.10 (4.11–6.63)	0.008
Blood gas analysis				
*PH*	7.40 (7.37–7.43)	7.40 (7.37–7.43)	7.40 (7.38–7.43)	0.636
*Pco_2_ (mmHg)*	49 (43–58)	49 (43–59)	48 (43–57)	0.901
*Po_2_ (mmHg)*	69 (57–80)	69 (57–80)	69 (57–80)	0.963
*Sao_2_*	94 (89–96)	94 (89–96)	94 (89–96)	0.912
Immunoglobulin				
*IgM (g/L)*	0.90 (0.60–1.21)	0.90 (0.62–1.22)	0.83 (0.60–1.11)	0.367
*IgA (g/L)*	2.04 (1.32–2.94)	1.99 (1.19–2.67)	2.31 (1.74–3.05)	0.035
*IgG (g/L)*	10.06 (8.50–13.60)	9.70 (8.30–12.20)	12.70 (10.80–14.90)	<0.001
*IgE (g/L)*	187 (71–594)	212 (84–676)	167 (69–318)	0.134
Sputum culture, n (%)				
*Positive sputum culture*	231 (34.7)	171 (32.2)	60 (44.8)	0.006
*Candida albicans*	65 (9.8)	53 (9.9)	12 (8.9)	0.721
*Pseudomonas aeruginosa*	39 (5.9)	13 (2.4)	26 (19.4)	<0.001
*Gram negative bacilli*	23 (3.5)	22 (4.1)	1 (0.7)	0.054

ALB – albumin, BCOS – bronchiectasis-chronic obstructive pulmonary disease overlap syndrome, COPD – chronic obstructive pulmonary disease, BUN – blood urea nitrogen, CRP – C-reactive protein, ESR – erythrocyte sedimentation rate, GLO – globulin, HB – haemoglobin, IgA – immunoglobulin A, IgE – immunoglobulin E, IgG – immunoglobulin G, IgM – immunoglobulin M, IQR – interquartile range, LNR – lymphocyte to neutrophil ratio, MD – median, Pco_2_ – partial pressure of carbon dioxide, PCT – procalcitonin, PH – the potential of hydrogen, PLT – platelets, Po_2_ – partial pressure of oxygen, Sao_2_ – arterial oxygen saturation, WBC – white blood cell

*Presented as MD (IQR) unless specified otherwise.

### Comparison of clinical characteristics and analysis of risk factors for acute exacerbation between AE+ and AE− groups

Comparison of the basic data of the AE+ and AE− groups revealed that patients in the AE+ group had a lower BMI, higher times of exacerbations in the past year, higher mMRC dyspnoea grade for admission, and a lower FVC (*P* < 0.05). There was no difference in the proportion of comorbidities between the two groups. Regarding medication, the proportion of patients using inhaled bronchodilators was significantly higher in the AE+ group (*P* < 0.05). There was no statistically significant difference in the proportion of patients using inhaled corticosteroids and systemic hormones (**Table 3**). When BCOS patients’ laboratory results were compared between the groups, it was found that the AE+ group had a greater lymphocyte-to-neutrophil ratio (LNR), CRP and ESR (*P* < 0.05). The AE+ group had increased sputum culture positivity and *Pseudomonas aeruginosa* culture positivity. Blood gas analysis, immunoglobulin levels, and hepatic and renal function did not differ statistically significantly between the two groups (**Table 4**).

**Table 3:** Comparison of baseline characteristics between AE+ and AE– groups*

Characteristics	BCOS (n = 118)	AE– (n = 50)	AE+ (n = 68)	*P*-value
Age in years	66 (59–71)	66 (58–73)	66 (61–71)	0.924
Male, n (%)	88 (74.6)	38 (76.0)	50 (73.5)	0.761
BMI (kg/m^2^)	21.30 (19.50–23.50)	22.20 (20.60–23.90)	20.80 (18.70–23.20)	0.019
Previous/current smokers, n (%)	74 (62.7)	31 (62.0)	43 (63.2)	0.891
Smocking pack-years	20 (0–40)	20 (0–40)	20 (0–40)	0.944
First diagnosis before 50 y old, n (%)	7 (5.9)	3 (6.0)	4 (5.9)	0.979
Hospitalisation days	10 (8–11)	10 (7–12)	9 (8–11)	0.893
Times of exacerbations in the past 12 mo	1 (1–2)	1 (1–1)	1 (1–2)	0.005
CAT score for admission	21 (17–25)	21 (16–25)	21 (18–26)	0.512
mMRC dyspnoea grade for admission	2 (2–3)	2 (2–3)	3 (2–3)	0.030
Improvements in CAT score	8 (4–12)	7 (3–12)	8 (5–12)	0.615
Improvements in mMRC dyspnoea grade	1 (0–1)	0 (0–1)	1 (0–1)	0.258
CAT score for discharge	14 (8–17)	14 (10–16)	13 (8–18)	0.444
mMRC dyspnoea grade for discharge	2 (1–2)	2 (1–2)	2 (1–2)	0.954
FEV1% predicted	34. 20 (26.40–45.00)	33.00 (25.40–47.80)	34.50 (26.40–44.70)	0.825
FVC (L)	1.94 (1.50–2.38)	2.28 (1.80–2.45)	1.89 (1.45–2.10)	0.009
FEV1/FVC	46.00 (34.00–61.00)	42.90 (35.20–65.90)	49.90 (31.00–60.70)	0.609
Comorbidities, n (%)				
*Hypertension*	35 (29.7)	15 (30.0)	20 (29.4)	0.945
*Diabetes mellitus*	13 (11.0)	5 (10.0)	8 (11.8)	0.762
*Obsolete pulmonary tuberculosis*	31 (26.3)	12 (24.0)	19 (27.9)	0.631
*Active pulmonary tuberculosis*	4 (3.4)	1 (2.0)	3 (4.4)	0.474
*Coronary artery disease*	18 (15.3)	9 (18.0)	9 (13.2)	0.477
*Arrhythmia*	7 (5.9)	3 (6.0)	4 (5.9)	0.979
*Pulmonary heart disease*	16 (13.6)	3 (6.0)	13 (19.1)	0.040
Medication				
*Inhalation of bronchodilators*	88 (74.6)	32 (64.0)	56 (82.4)	0.024
*ICS*	58 (49.2)	24 (48.0)	34 (50.0)	0.830
*Systemic hormone*	11 (9.3)	7 (14.0)	4 (5.9)	0.134

AE – acute exacerbation, AE+ – acute exacerbation occurred during the follow-up period, AE– – no acute exacerbation occurred during the follow-up period, BCOS – bronchiectasis-chronic obstructive pulmonary disease overlap syndrome, BMI – body mass index, CAT – chronic obstructive pulmonary disease assessment test, FEV1 – forced expiratory volume in one second, FVC – forced vital capacity, ICS – inhaled corticosteroids, IQR – interquartile range, MD – median, mMRC – modified Medical Research Council

*Presented as MD (IQR) unless specified otherwise.

**Table 4:** Comparison of laboratory examination between AE– and AE+ groups*

Laboratory examinations	BCOS (n = 118)	AE– (n = 50)	AE+ (n = 68)	*P*-value
Blood routine				
*WBC ( × 10^9^/L)*	6.99 (5.47–9.02)	6.99 (5.72–8.71)	6.87 (5.44–9.02)	0.365
*HB (g/L)*	131 (117–140)	135 (116–143)	131 (119–139)	0.492
*PLT ( × 10^9^/L)*	237 (181–294)	245 (177–318)	237 (182–278)	0.618
*Neutrophils ( × 10^9^/L)*	4.90 (3.90–7.10)	4.90 (4.10–7.10)	4.90 (3.40–6.80)	0.341
*Lymphocytes ( × 10^9^/L)*	1.19 (0.89–1.46)	1.14 (0.80–1.46)	1.22 (1.00–1.44)	0.508
*Eosinophils ( × 10^9^/L)*	0.15 (0.06–0.25)	0.16 (0.05–0.29)	0.14 (0.07–0.23)	0.715
*Eosinophils ≥0.3 × 10^9^/L, n (%)*	26 (22.0)	12 (24.0)	14 (20.6)	0.659
*LNR*	0.25 (0.17–0.32)	0.23 (0.17–0.29)	0.15 (0.18–0.38)	0.042
Inflammatory markers				
*CRP (mg/L)*	7.50 (3.50–16.90)	4.80 (3.10–11.70)	9.90 (4.30–20.80)	0.006
*ESR (mm/h)*	22 (7–45)	13 (7–29)	25 (11–53)	0.032
*PCT (mg/L)*	0.10 (0.05–0.11)	0.10 (0.05–0.12)	0.07 (0.05–0.10)	0.201
Hepatic and renal function				
*ALB (g/L)*	35.00 (32.30–37.70)	35.80 (31.10–37.70)	35.00 (32.50–37.70)	0.851
*GLO (g/L)*	27.20 (24.60–31.10)	27.10 (24.60–30.70)	27.80 (24.90–31.20)	0.537
*Creatinine (μmol/L)*	65.30 (56.40–76.60)	65.40 (58.20–78.70)	65.10 (56.10–75.80)	0.750
*BUN (mmol/L)*	4.80 (3.90–6.20)	5.10 (3.70–6.10)	4.80 (4.10–6.20)	0.963
Blood gas analysis				
*PH*	7.40 (7.38–7.43)	7.40 (7.38–7.44)	7.40 (7.37–7.42)	0.254
*Pco_2_ (mmHg)*	48 (43–56)	49 (43–54)	47 (43–58)	0.991
*Po_2_ (mmHg)*	70 (58–82)	69 (60–80)	71 (57–83)	0.585
*Sao_2_*	94 (90–96)	94 (91–96)	94 (89–96)	0.844
Immunoglobulin				
*IgM (g/L)*	0.81 (0.65–1.11)	0.88 (0.55–1.11)	0.81 (0.68–1.11)	0.983
*IgA (g/L)*	2.02 (1.66–2.95)	2.65 (1.74–4.16)	1.93 (1.58–2.94)	0.182
*IgG (g/L)*	12.20 (10.80–14.10)	12.10 (9.11–14.10)	12.70 (11.20–14.90)	0.667
*IgE (g/L)*	183 (71–403)	172 (65–213)	190 (108–403)	0.259
Sputum culture, n (%)				
*Positive sputum culture*	40 (43.5)	16 (38.1)	24 (48.0)	0.03
*Candida albicans*	7 (7.6)	4 (9.5)	3 (6.0)	0.525
*Pseudomonas aeruginosa*	17 (18.5)	4 (9.5)	13 (26.0)	0.043)
*Gram negative bacilli*	4 (4.3)	2 (4.8)	2 (4.0)	0.858

AE – acute exacerbation, AE+ – acute exacerbation occurred during the follow-up period, AE– – no acute exacerbation occurred during the follow-up period, ALB – albumin, BCOS – bronchiectasis-chronic obstructive pulmonary disease overlap syndrome, BUN – blood urea nitrogen, CRP – C-reactive protein, ESR – erythrocyte sedimentation rate, GLO – globulin, HB – haemoglobin, IgA – immunoglobulin A, IgE – immunoglobulin E, IgG – immunoglobulin G, IgM – immunoglobulin M, IQR – interquartile range, LNR – lymphocyte to neutrophil ratio, MD – median, Pco_2_ – partial pressure of carbon dioxide, PCT – procalcitonin, PH – the potential of hydrogen, PLT – platelets, Po_2_ – partial pressure of oxygen, Sao_2_ – arterial oxygen saturation, WBC – white blood cell

*Presented as MD (IQR) unless specified otherwise.

**Figure 2:**
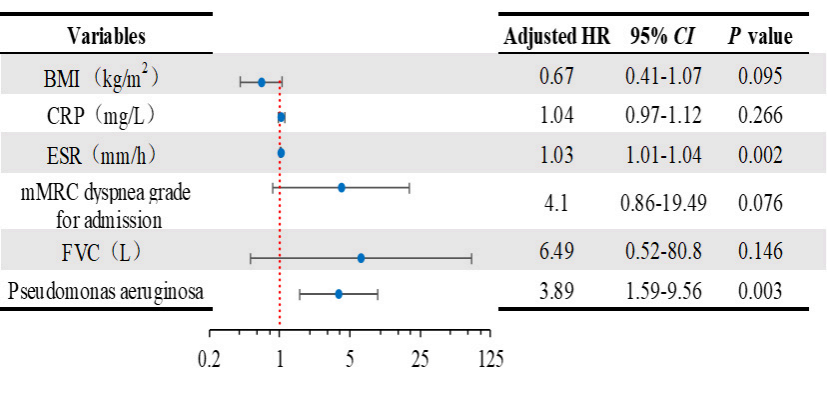
Regression analysis of potential factors related to acute exacerbation in BCOS patients. BMI – body mass index, CI – confidence interval, CRP – C-reactive protein, ESR – erythrocyte sedimentation rate, FCV – forced vital capacity HR – hazard ratio, mMRC – modified Medical Research Council.

BMI, CRP, ESR, mMRC dyspnoea grade for admission, FVC at baseline and *Pseudomonas aeruginosa* culture positivity were associated with a higher risk of severe acute exacerbation in univariate analysis (*P* < 0.05). Both ESR at baseline (hazard ratio (HR) = 1.03; 95% confidence interval (CI) = 1.01–1.04, *P* = 0.002) and *Pseudomonas aeruginosa* culture positivity (HR = 3.89; 95% CI = 1.59–9.56, *P* = 0.003) were confirmed as independent predictors of severe acute exacerbation in multivariate analysis during the years of follow-up (**Figure 2**). There was no difference in risk of acute exacerbation associated with the BMI, CRP, mMRC dyspnoea grade for admission and FVC.

### Comparison of clinical characteristics and analysis of risk factors for death between patients in the survival and death group

When we compared the basic clinical data from the survival and death groups, we found that the patients in the death group were statistically significantly older, had a lower BMI, spent longer time in the hospital, had a higher mMRC dyspnoea grade and CAT scores upon admission and discharge, had a higher rate of comorbidities with obsolete pulmonary tuberculosis and pulmonary heart disease, and used inhaled bronchodilators less frequently than the patients in the survival group (*P* < 0.05). When comparing the pulmonary function indicators of the two groups, there was no discernible change (**Table 5**). Laboratory test results comparing the two groups showed that patients in the death group had higher values of procalcitonin and BUN, a higher percentage of sputum cultures positive for fungi, and lower levels of haemoglobin, PLT, lymphocyte count, LNR, albumin, and arterial oxygen saturation (*P* < 0.05). The immunoglobulin levels in the two groups were identical (**Table 6**).

**Table 5:** Comparison of baseline characteristics between death and survival groups*

Characteristics	BCOS (n = 171)	Death group (n = 32)	Survival group (n = 139)	*P*-value
Age in years	67 (61–72)	70 (65–75)	66 (60–71)	0.010
Male, n (%)	133 (77.8)	27 (84.4)	106 (76.3)	0.319
BMI (kg/m^2^)	20.90 (18.60–23.50)	18.70 (16.80–22.30)	21.20 (19.30–23.60)	0.022
Previous/current smokers, n (%)	110 (64.3)	21 (65.6)	89 (64)	0.865
Smocking pack-years	20 (0–40)	30 (0–40)	20 (0–40)	0.892
First diagnosis before 50 y old, n (%)	11 (6.4)	3 (9.4)	8 (5.8)	0.452
Hospitalisation days	10 (8–12)	12 (9–15)	10 (8–11)	0.003
Times of exacerbations in the past 12 mo	1 (1–3)	2 (1–3)	1 (1–2)	0.192
CAT score for admission	22 (17–27)	26 (18–31)	21 (17–26)	0.038
mMRC dyspnoea grade for admission	3 (2–4)	3 (3–4)	3 (2–3)	0.013
Improvements in CAT score	7 (3–12)	7 (6–8)	7 (3–12)	0.464
Improvements in mMRC dyspnoea grade	1 (0–1)	0 (0–1)	1 (0–1)	0.260
CAT score for discharge	14 (10–20)	22 (14–25)	14 (9–18)	0.006
mMRC dyspnoea grade for discharge	2 (1–3)	3 (3–4)	2 (1–2)	<0.001
FEV1% predicted	33.40 (26.40–44.70)	31.70 (25.50–41.70)	33.90 (26.80–45.00)	0.471
FVC (L)	1.94 (1.43–2.38)	1.64 (1.34–2.16)	1.96 (1.45–2.39)	0.152
FEV1/FVC	43.40 (34.20–60.00)	43.30 (37.50–53.00)	43.50 (33.90–60.20)	0.898
Comorbidities, n (%)				
*Hypertension*	49 (28.7)	10 (31.3)	39 (28.1)	0.719
*Diabetes mellitus*	20 (11.7)	4 (12.5)	16 (11.5)	0.875
*Obsolete pulmonary tuberculosis*	52 (30.4)	17 (53.1)	35 (25.2)	0.002
*Active pulmonary tuberculosis*	6 (3.5)	1 (3.1)	5 (3.6)	0.896
*Coronary artery disease*	23 (13.5)	3 (9.4)	20 (14.4)	0.454
*Arrhythmia*	11 (6.4)	3 (9.4)	8 (5.8)	0.452
*Pulmonary heart disease*	32 (18.7)	13 (40.6)	19 (13.7)	<0.001
Medication				
*Inhalation of bronchodilators*	119 (69.6)	17 (53.1)	102 (73.4)	0.025
*ICS*	83 (48.5)	14 (43.8)	69 (49.6)	0.548
*Systemic hormone*	150 (87.7)	26 (81.3)	124 (89.2)	1.529

BCOS – bronchiectasis-chronic obstructive pulmonary disease overlap syndrome, BMI – body mass index, CAT – chronic obstructive pulmonary disease assessment test, FEV1 – forced expiratory volume in one second, FVC – forced vital capacity, ICS – inhaled corticosteroids, IQR – interquartile range, MD – median, mMRC – modified Medical Research Council

*Presented as MD (IQR) unless specified otherwise.

**Table 6:** Comparison of laboratory examination between death and survival groups*

Laboratory examinations	BCOS (n = 171)	Death group (n = 32)	Survival group (n = 139)	*P*-value
Blood routine				
*WBC ( × 10^9^/L)*	7.04 (5.51–9.07)	7.04 (5.80–8.61)	7.11 (5.51–9.07)	0.851
*HB (g/L)*	129 (117–140)	120 (107–131)	132 (118–140)	0.014
*PLT ( × 10^9^/L)*	226 (175–289)	187 (151–259)	238 (181–293)	0.044
*Neutrophils ( × 10^9^/L)*	5.20 (3.90–7.20)	5.60 (3.90–7.50)	4.90 (3.90–7.10)	0.242
*Lymphocytes ( × 10^9^/L)*	1.16 (0.87–1.42)	1.02 (0.66–1.24)	1.22 (0.94–1.48)	0.002
*Eosinophils ( × 10^9^/L)*	0.15 (0.06–0.25)	0.11 (0.04–0.17)	0.15 (0.06–0.26)	0.060
*Eosinophils ≥0.3 × 10^9^/L, n (%)*	94 (54.9)	14 (43.7)	80 (57.6)	0.157
*LNR*	0.23 (0.14–0.32)	0.17 (0.11–0.22)	0.25 (0.16–0.33)	0.002
Inflammatory markers				
*CRP (mg/L)*	9.20 (3.90–26.80)	14.60 (6.40–53.40)	8.80 (3.60–24.70)	0.055
*ESR (mm/h)*	25 (8–48)	34 (10–45)	24 (7–50)	0.528
*PCT (mg/L)*	0.10 (0.05–0.12)	0.11 (0.05–0.16)	0.10 (0.05–0.11)	0.010
Hepatic and renal function				
*ALB (g/L)*	34.20 (31.30–37.20)	31.20 (29.00–34.80)	34.80 (32.10–37.60)	<0.001
*GLO (g/L)*	27.60 (24.90–31.10)	28.50 (25.20–30.60)	27.40 (24.60–31.20)	0.648
*Creatinine (μmol/L)*	65.40 (56.00–76.70)	63.30 (50.10–82.80)	65.80 (56.20–76.30)	0.761
*BUN (mmol/L)*	5.10 (4.10–6.60)	7.30 (5.60–8.70)	4.80 (3.90–6.30)	<0.001
Blood gas analysis				
*PH*	7.40 (7.38–7.43)	7.39 (7.37–7.42)	7.40 (7.38–7.43)	0.437
*Pco_2_ (mmHg)*	48 (43–57)	52 (45–65)	48 (43–56)	0.054
*Po_2_ (mmHg)*	69 (57–80)	64 (52–75)	71 (58–82)	0.063
*Sao_2_*	94 (90–96)	92 (88–95)	94 (90–96)	0.025
Immunoglobulin				
*IgM (g/L)*	0.83 (0.60–1.11)	0.54 (0.46–0.83)	0.83 (0.68–1.14)	0.155
*IgA (g/L)*	2.31 (1.74–3.05)	2.37 (1.88–4.01)	2.28 (1.71–3.05)	0.414
*IgG (g/L)*	12.70 (10.80–14.90)	14.70 (12.30–16.60)	12.20 (10.80–14.30)	0.233
*IgE (g/L)*	167 (69–318)	167 (71–269)	172 (69–318)	0.818
Sputum culture, n (%)				
*Positive sputum culture*	60 (44.8)	16 (59.20)	44 (41.1)	0.090
*Candida albicans*	12 (8.9)	5 (18.5)	7(6.5)	0.051
*Pseudomonas aeruginosa*	26 (19.4)	8 (29.6)	18 (16.8)	0.133
*Gram negative bacilli*	5 (3.7)	3 (11.1)	2 (1.9)	0.024

ALB – albumin, BCOS – bronchiectasis-chronic obstructive pulmonary disease overlap syndrome, BUN – blood urea nitrogen, CRP – C-reactive protein, ESR – erythrocyte sedimentation rate, GLO – globulin, HB – haemoglobin, IgA – immunoglobulin A, IgE – immunoglobulin E, IgG – immunoglobulin G, IgM – immunoglobulin M, IQR – interquartile range, LNR – lymphocyte to neutrophil ratio, MD – median, Pco_2_ – partial pressure of carbon dioxide, PCT – procalcitonin, PH – the potential of hydrogen, PLT – platelets, Po_2_ – partial pressure of oxygen, Sao_2_ – arterial oxygen saturation, WBC – white blood cell

*Presented as MD (IQR) unless specified otherwise.

**Figure 3:**
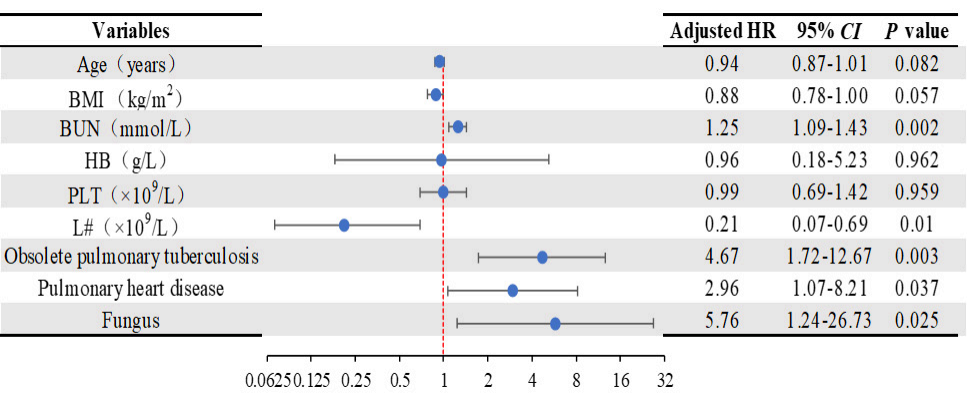
Regression analysis of potential factors related to death in BCOS patients. BMI – body mass index, BUN – blood urea nitrogen, CI – confidence interval, HB – haemoglobin, L# – lymphocyte count, PLT – platelets.

Age, BMI, BUN, haemoglobin, PLT, lymphocyte count, comorbidities with obsolete pulmonary tuberculosis, comorbidities with pulmonary heart disease and fungus culture positivity were associated with a higher risk of severe acute exacerbation in univariate analysis (*P* < 0.05). Fungus culture positivity (HR = 5.76; 95% CI = 1.24–26.73, *P* = 0.025), baseline BUN (HR = 1.25; 95% CI = 1.09–1.43, *P* = 0.002), reduced baseline lymphocyte count (HR = 0.21; 95% CI = 0.07–0.69, *P* = 0.01), comorbidities with obsolete pulmonary tuberculosis (HR = 4.67; 95% CI = 1.72–12.67, *P* = 0.003) and comorbidities with pulmonary heart disease (HR = 2.96; 95% CI = 1.07–8.21, *P* = 0.037) were verified as independent predictors of death in multivariate analysis during the years of follow-up (**Figure 3**). There was no difference in risk of death associated with age, BMI, haemoglobin, and PLT.

**Figure 4:**
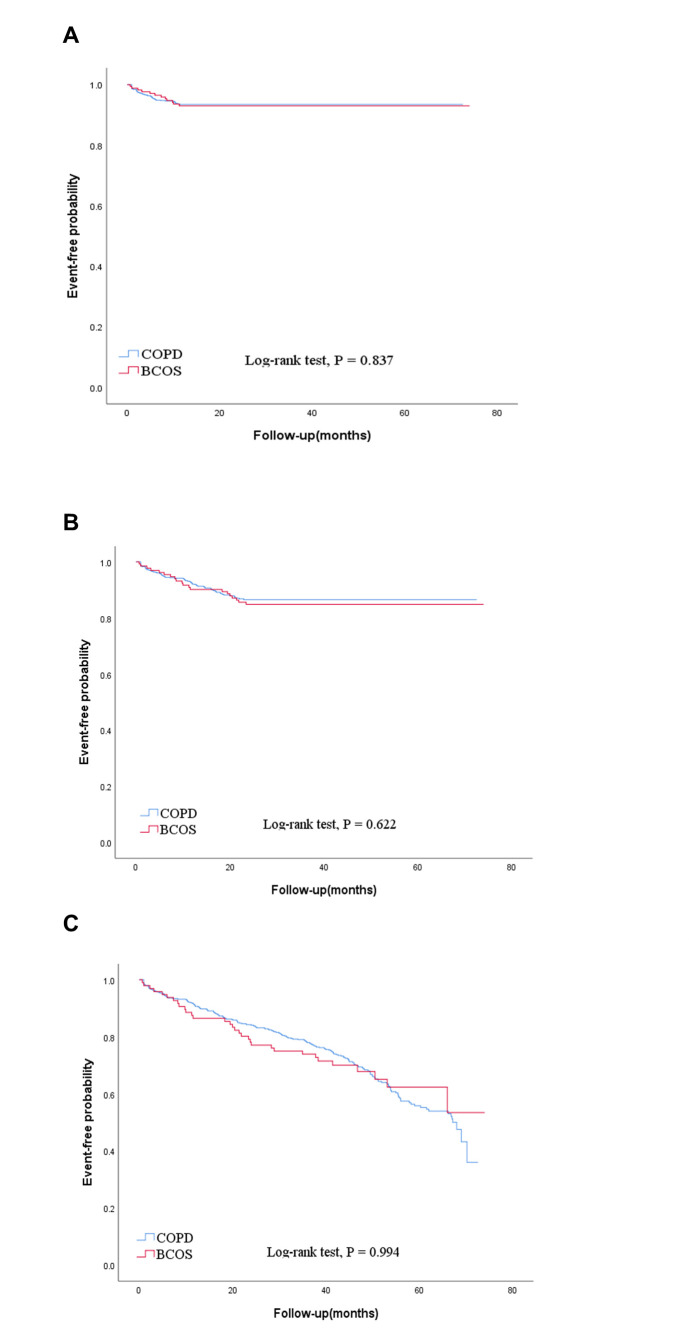
Kaplan-Meier curves for time to death for patients in groups COPD and BCOS. **Panel A.** The first year of follow-up. **Panel B.** Second year of follow-up. **Panel C.** Third year of follow-up.

After one, two, and three years of follow-up, we compared the previous year’s mortality and severe acute exacerbations between the COPD g and the BCOS groups. The findings indicated no statistically significant difference in mortality or severe acute exacerbation during the follow-up period between the two groups (**Table 7**). Kaplan-Meier curves under survival analysis demonstrated no statistically significant difference in mortality between the COPD and BCOS groups at the full one (*P* = 0.837), two (*P* = 0.622), and three (*P* = 0.994) years of follow-up (**Figure 4**).

**Table 7:** Comparison of follow-up results between COPD and BCOS groups

Items	Total	COPD	BCOS	*P*-value
One year follow-up, n	875	704	171	
*Death, n (%)*	58 (6.6)	46 (6.5)	12 (7.0)	0.837
*Times of severe exacerbations in the past 12 mo, MD (IQR)*	1 (0–2)	1 (0–2)	1 (0–2)	0.539
Two years follow-up, n	786	654	132	
*Death, n (%)*	108 (13.7)	88 (13.5)	20 (15.2)	0.622
*Times of severe exacerbations in the past 12 mo, MD (IQR)*	1 (0–2)	1 (0–2)	1 (0–2)	0.582
Three years follow-up, n	654	558	96	
*Death, n (%)*	233 (35.6)	201 (36.0)	32 (33.3)	0.944
*Times of severe exacerbations in the past 12 mo, MD (IQR)*	1 (0–2)	1 (0–2)	1 (0–2)	0.890

BCOS – bronchiectasis- chronic obstructive pulmonary disease overlap syndrome, COPD – chronic obstructive pulmonary disease, MD – median, IQR – interquartile range

## DISCUSSION

This was a real-world, observational, prospective clinical study designed to explore the clinical characteristics, prognosis, and factors influencing the outcome of patients with BCOS. First, we discovered that patients in the BCOS group had lower symptom scores, worse lung function, a higher likelihood of co-occurring active tuberculosis, higher levels of eosinophils and inflammatory markers, and a higher rate of positive sputum cultures for *Pseudomonas aeruginosa* than patients in the COPD group. We looked more closely at the clinical traits and related risk factors of the BCOS group patients who had suffered from severe acute exacerbations. Patients in the AE+ group were found to have lower BMI, higher symptom scores, higher inflammatory markers, poorer lung function markers, higher rates of using inhaled bronchodilators, and higher rates of both positive and *Pseudomonas aeruginosa* positive sputum cultures. Among these, *Pseudomonas aeruginosa*-positive sputum cultures and ESR were independent risk factors for the incidence of acute exacerbations in BCOS patients. We explored the clinical characteristics and associated risk factors of BCOS group patients who passed away during follow-up. Patients in the death group were found to be older, have a lower BMI, spent a longer time in the hospital, had higher symptom scores, higher levels of inflammatory markers, higher blood pressure, lower rates of using inhaled bronchodilators, were more likely to have a combination of pulmonary heart disease and obsolete pulmonary tuberculosis and to had a higher rate of fungus-positive sputum cultures. Fungus-positive sputum cultures, BUN, lymphocyte count, and comorbidities with pulmonary heart disease and obsolete pulmonary tuberculosis were independent risk factors for the incidence of death in BCOS patients.

Blood eosinophil counts (BECs) have garnered attention in bronchiectasis due to the growing emphasis on them as a biomarker in asthma and COPD. After analysing sputum and BECs from 1007 patients across five countries, Shoemark et al. discovered that eosinophil counts in sputum were connected with BEC levels (correlation coefficient (r) = 0.31; *P* < 0.001) and that approximately 20% of patients with bronchiectasis had high BEC levels (BEC>300/μl) [17]. Sputum eosinophil count and BEC levels corresponded (r = 0.31; *P* < 0.001), leading to the theory that elevated BEC levels could serve as a stand-in for airway eosinophil counts. Similar to what we saw in the COPD group, patients with BCOS had considerably higher blood eosinophil counts, indicating that BCOS is most likely an inflammatory response mediated by eosinophils. According to BEC values, another study aimed to evaluate the impact of inhaled corticosteroids on different outcomes in patients with BCOS [18]. The findings showed that the annual rate of hospitalisations and exacerbations increased with increasing BECs. Pneumonic episodes, chronic bronchial infections caused by pathogenic microorganisms, and incident exacerbations were more common in patients with BEC<100/μL. The number of exacerbations (1.77 vs 1.08; *P* < 0.001), and their severity (0.67 vs 0.35; *P* = 0.011) were only reduced in eosinophilia patients (>300 eosinophils/μL) treated with inhaled corticosteroids.

The most prevalent IgG subclass defects in patients with branchial expansion are immunoglobulin G2 (IgG2) defects. Zhang et al. discovered that lower IgG2 levels were an independent risk factor for exacerbations of branchial expansion and that patients with lower IgG2 levels (≤3.53 g/L) had higher bronchiectasis severity index scores than patients with higher IgG2 levels (>4.45 g/L) (*P* = 0.013 and *P* = 0.003, respectively) [19]. According to certain research, older persons with COPD who experience acute exacerbations of the disease have significantly higher levels of IgA, IgG, and immunoglobulin M than older adults without the disease. Patients also exhibit aberrant humoral immune activity [20]. The BCOS group in our study had greater IgA and IgG values than the COPD group, indicating that humoral immune activity is hyperactive in BCOS patients. However, the British Thoracic Society guidelines for branchial enlargement do not advise frequent testing for IgG subclasses, and further study is required to bolster this conclusion.

*Pseudomonas aeruginosa* is one of the main pathogens involved in bronchiectasis, and its infection is a major factor in developing the disease. For this reason, studying the microorganisms in the airways of patients suffering from bronchiectasis will be useful in determining the course of treatment and forecasting the subsequent development of the disease. Interleukin-1β is a pro-inflammatory cytokine activated by the Nod-like receptor, pyrin domain containing three and Nod-like receptor, containing four inflammatory complex. Yang et al. discovered that the innate immunity factor, interferon-beta, promotes the cytoplasmic survival of *Pseudomonas aeruginosa* in macrophages and that its production is inhibited [21]. These findings establish a connection between innate immunity and *Pseudomonas aeruginosa*. Consistent with earlier research, our analysis revealed that patients in the BCOS group had a higher likelihood of concurrent *Pseudomonas aeruginosa* infections than COPD patients, as well as during acute exacerbations. Some studies have found that infection by potentially pathogenic microorganisms is involved in the main pathomechanisms of BCOS, with *Pseudomonas aeruginosa* being the pathogen with the highest frequency. It has been shown that *Pseudomonas aeruginosa* is a biomarker for BCOS as it can cause airway and systemic inflammatory responses and lead to lung function impairment [22,23].

Although secondary bronchiectasis is common in patients with COPD, little is known about the clinical consequences and medical expenses of bronchiectasis in combination with COPD. According to recent research, individuals with BCOS, compared to those with bronchiectasis or COPD alone, have greater rates of allergic bronchopulmonary aspergillosis (ABPA) and higher bronchiectasis severity index [24]. Additionally, our research revealed that positive fungal cultures were a separate risk factor for death in BCOS patients and that patients who died from the disease were more likely to have concurrent fungal infections. Furthermore, compared to patients receiving ABPA alone, those receiving ABPA with BCOS showed worse lung function as well as more frequent and severe exacerbations. As a result, medical professionals need to be aware that BCOS patients with a high bronchiectasis severity index may have ABPA.

Bronchiectasis develops and progresses due to a vicious cycle that includes bacterial colonisation of COPD, airway inflammation, and structural destruction of the airways [25]. This loop impairs intrinsic and adaptive immunity in the airways [26]. Our research indicates that airway inflammation is always present in the evolution of BCOS, as evidenced by the significantly higher inflammatory indexes of the patients in both the group with basic BCOS and the group with acute aggravation or death of BCOS. As a result, patients with BCOS should focus especially on enhancing the body’s general immune system and airway clearance capabilities.

Up until the current follow-up period, our study did not find any statistically significant differences between BCOS and COPD patients’ rates of acute exacerbations or mortality. Studies examining the relationship between acute exacerbation and mortality in individuals with BCOS that have a long-term follow-up are non-existent. We hypothesise that this is because the majority of patients with pure COPD that we included were hospitalised patients experiencing an acute exacerbation; in addition to bronchiectasis, which can lead to recurrent infections, COPD itself has structural alterations in the airways, such as emphysema and alveoli, which are also highly susceptible to concurrent infections that worsen the condition and have a poor prognosis [27,28]. Therefore, there is unlikely to be a significant difference between the two. Still, we also need to extend the follow-up time and investigate the long-term survival of patients in the BCOS group.

Our study has several limitations. First, our cohort was not population-based; patients were recruited at a tertiary hospital in China. Therefore, the results may not be generalisable to all Chinese BCOS patients. However, our findings have important clinical implications and objectively evaluated the situation in Hunan, reflecting BCOS patients’ current clinical characteristics and prognosis status. Long-term follow-up data of patients elsewhere will be collected and analysed in our future studies. Second, the small sample size of the BCOS death group and the small number of deaths during follow-up limited our ability to find potentially significant associations. Third, confounders from unmeasured variables, such as comorbidities and medication treatment during follow-up, could also influence clinical outcomes. Finally, future studies should focus on other factors that may influence the clinical characteristics of patients with BCOS.

## CONCLUSIONS

Patients with BCOS present with reduced lung function, increased susceptibility to different complications, elevated blood eosinophils and inflammatory markers, and elevated rates of positive *Pseudomonas aeruginosa* cultures. These distinctive markers are linked to a greater risk of severe acute exacerbations and mortality.
